# Self-reported mental health outcomes of International medical graduates in Australia: a cross-sectional survey

**DOI:** 10.3389/fmed.2025.1615471

**Published:** 2025-08-07

**Authors:** Sunita Joann Rebecca Healey, Kristy Fakes, Bunmi S. Malau-Aduli, Lucy Leigh, Balakrishnan R. Nair

**Affiliations:** ^1^School of Medicine and Public Health, University of Newcastle, Callaghan, NSW Australia; ^2^Equity in Health and Wellbeing Research Program, Hunter Medical Research Institute, New Lambton Heights, NSW, Australia; ^3^Center for Medical Professional Development, Hunter New England Local Health District, New Lambton Heights, NSW, Australia; ^4^Heart and Stroke Research Program, Hunter Medical Research Institute, New Lambton Heights, NSW, Australia; ^5^College of Medicine and Dentistry, James Cook University, Townsville, QLD, Australia; ^6^Data Sciences, Hunter Medical Research Institute, New Lambton Heights, NSW, Australia

**Keywords:** wellbeing, burnout, migrant, workplace, discrimination, foreign medical graduate

## Abstract

**Introduction:**

International medical graduates are an important migrant workforce with unique challenges which may compound mental health outcomes. We examined the rates of self-reported wellbeing, psychological distress and burnout by IMGs in Australia by undertaking a cross-sectional survey of IMGs.

**Methods:**

In late 2023, an online survey of three validated self-reporting mental health instruments was distributed non-randomly to IMGs across Australia, to identify symptoms of wellbeing, likelihood of psychological distress, and burnout.

**Results:**

Of the 286 participants who started the survey, 199 completed the Wellbeing instrument, 191 completed the Kessler (K6) instrument, and 181 completed the Burnout instrument. The calculated wellbeing mean score of participants was 54.6/100 [SD 23.18; median score: 80/100 (27 participants); range: 0–100]. 30/191 (15.7%) participants recorded a K6 score between 19 and 30, indicating a high likelihood of serious psychological distress. 84/181 (46.4%) participants recorded a score indicating some level of burnout. Statistically significant associations (*p* < 0.001) between ‘Wellbeing’ and ‘Burnout’ versus “Discrimination experienced in the last 5 years” were identified.

**Discussion:**

IMGs may be at risk of poor mental health outcomes resulting from their unique experiences, including perceived discrimination. Further exploration in larger and more robust studies is recommended to confirm preliminary findings and address challenges faced by this important migrant workforce.

## Introduction

International medical graduates are a vital migrant workforce worldwide. IMGs are doctors who have obtained their primary medical qualification (PMQ) from a different country where they are working or residing. Accounting for approximately 30% of doctors in many developed countries such as Australia, IMGs are commonly recruited to fill workforce shortages and are therefore disproportionately represented in areas of geographical isolation or service positions of need (1, 2). IMGs may experience challenges related to migration, employment and cultural shifts to a new country (3). It is important for institutions to be mindful of stressors impacting critical workers, such as IMGs, to ensure health workforce security.

“Mental health” encompasses a broad spectrum of conditions, from wellbeing and satisfaction, through to psychiatric disorders (4). Mental health is defined by the World Health Organization, as “…a state of mental wellbeing that enables people to cope with the stresses of life, realize their abilities, learn well and work well, and contribute to their community” (5).

Doctors are at risk of poor mental health, due to demanding workloads and onerous responsibilities, often coupled with competitive and perfectionist tendencies (4, 6). Long hours, isolation in rural placements and uncertainty of future work locations have been identified as additional stressors for doctors (4, 7).

Numerous studies report higher rates of mental health disorders in doctors when compared to the general population (4, 8). Burnout has been reported as high as 68% in some international studies and suicide rates are consistently reported above the general population (9, 10). Anxiety, depression and substance misuse are also well-described (10, 11).

Understanding the mental health problems encountered by doctors is essential for optimizing adequate and safe clinical care, productivity and workforce sustainability. Poorer rates of job satisfaction have been linked to higher turnover rates, which may contribute to disrupted patient care and affect job satisfaction for the remaining staff (12, 13).

International medical graduates face additional challenges such as adaptation to a new healthcare system, differences in communication styles and patient interactions, financial costs associated with examinations and relocation, rural living and workplace discrimination (14–17). A recent Commission report by the World Psychiatric Association highlighted concerns for IMG mental health and the lack of published data (18).

We aimed to explore the self-reported wellbeing and rates of burnout and psychological distress amongst IMGs in Australia. We were also interested to identify if there was any association between reported adverse mental health outcomes and other items, such as demographics or report of discrimination in the last 5 years. The study formed part of a larger Ph.D body of work, broadly investigating the journeys and lived experiences of IMGs in Australia.

## Materials and methods

### Design and setting

The survey was designed as a cross-sectional, observational study of IMGs. The online survey was distributed to IMGs across Australia. A fully online format was chosen to maximize completion rates, maintenance of data integrity and user friendliness. The survey was active for 11 weeks, between 13th October 2023 and 31 December 2023. The REDCap (Research Electronic Data Capture) tool, hosted by Hunter Medical Research Institute, was used to create the survey and manage the data (19, 20). REDCap is a secure data capture platform which is used for surveys in health sciences research. The study was approved by College Human Ethics Advisory Panel University of Newcastle as low risk research: H2022-0392; with Access Request granted through Hunter New England Health Human Research Ethics Committee (HREC): AR20230405_Nair and Central Coast Health HREC: 0323-024C.

### Recruitment and participants

Any medical doctor with a foreign PMQ (e.g., MBBCh, MBBS etc.) currently residing in Australia, was eligible for participation, irrespective of current employment status.

The online survey invitation was distributed to a non-random sample of IMGs based in Australia, identified via existing personal and professional networks. Networks included public sector health workplaces, a national post-graduate training college, IMG social media and support groups and unofficial groups of IMG community members. The customized survey link was circulated by email, social media, text message and flyer (QR code), facilitated by snowball recruitment. We used various recruitment avenues, to reduce selection bias and improve opportunity to capture a variety of unique IMG journeys across the country. Details of recruiting networks are deliberately unnamed to protect the identity of participants.

### Survey instruments

Three validated and widely used mental health instruments were chosen to identify for symptoms of wellbeing, psychological distress and burnout: the WHO 5-item wellbeing instrument (21), the Kessler 6 (K6) (22, 23), and a single-item instrument for physician burnout (24). The WHO 5-item wellbeing instrument is a short 5-item rating scale subjectively measuring wellbeing, where respondents indicate frequency of wellbeing items (e.g., “I have felt fresh and rested” over the past 2 weeks) (21). Respondents’ raw scores between 0 (at no time) and 5 (all the time) are then transferred to 0–100 score; where 0 represents the worst and 100 represents the best imaginable wellbeing (21). The K6 measures self-reported frequency of psychological distress symptoms (e.g., worthlessness) over the past 30 days (22). Using the Australian Bureau of Statistics (2012) scoring system, respondents indicated frequency of symptoms from 1 to 5: none of the time, a little of the time, some of the time, most of the time and all of the time (23). K6 scores totaling 19–30 indicated a high likelihood or risk of having serious psychological distress, whilst low likelihood or risk of having serious psychological distress was indicated by total K6 scores ≤ 18. A recent systematic review examining 17 studies of physicians and trainees found that the single-item instrument for burnout showed statistically significant and adequate reliability for predicting the emotional exhaustion component of the Maslach Burnout Inventory *r* = 0.71 (95% CI 0.67–0.74; I^2^ = 89%) (25). The single-item instrument has been used in other Australian studies, examining burnout in Australian clinical cancer workers and GP registrars (26, 27) Our study respondents were asked to choose from descriptive Likert-style scoring options ranging from 1 to 6 which best described their current situation. Options 1 and 2 indicated no burnout, and options 3–6 indicated burnout. These three instruments were chosen based on their suitability to address our study aims and suit our population i.e., user friendliness and brevity. Demographics were chosen as important by the multidisciplinary research team, based on results of an earlier scoping review (14) and to address the overarching research focus. A draft copy of the survey underwent multiple iterations, including review by a team of six IMGs plus two experts in quantitative research and a statistician; and a final test by ten IMG respondents within the 2 months prior to national distribution of the final version.

### Data collection

The two screening questions alone were compulsory. All other questions were deliberately left optional, to maximize progression and participation across all sections of the survey. Hence, differences in participation rates may be seen within the items reported in the “Section Results.” The estimated duration of 20 min completion time was forewarned to participants in the Participant Information Statement. A variety of formats were delivered throughout the survey, to maintain participant interest and gather significant details, e.g., single/multiple answer, binary, Likert scales and open-ended options. All data was collected anonymously.

### Sample size

Based on expected consent rates and eligibility, we calculated that a minimum sample size of 200 survey participants would enable estimation of the proportion of participants reporting experiences with 95% confidence intervals with a precision of ± 4.7%. A power calculation for specific hypothesis testing was not possible, due to the inability to freely access data reporting the precise total number of IMGs in Australia.

### Data analysis

The data was analysed using STATA version 17 (28). The characteristics of the sample (describing demographics and experiences) were explored via descriptive statistics [mean (SD) for continuous variables, *N* (%) for categorical].

Participants’ responses were cleaned, and variables were re-coded to simplify analysis and thereby provide more meaningful results based on the sample size. For example, di- or trichotomized numerous variables: gender (male vs. female), marital status (married/*de facto* vs. Other), ethnicity [British/Irish vs. European (including Eastern European) vs. all other], native language (English vs. all other), religion (religion vs. no religion), PMQ country (graduates from ‘competent authority pathway’ countries vs. all other countries), employment status (full/part-time work vs. non-working i.e., leave/retired/volunteer/disability pension), training status (currently training vs. not), work region (metropolitan only vs. rural/remote/mixed) and registration (full/unconditional vs. provisional, limited or non-practicing vs. none). Marital status and religion were dichotomized as surrogate markers for support. Language was dichotomized as surrogate markers of privilege of ethnicity and language. PMQ country was dichotomized as graduates from Canada, USA, UK, Ireland and New Zealand are exempted from standard examination processes, as per the ‘competent authority pathway’ granted by the Australian Medical Council. For the purposes of data analysis, low response rates were recoded to ‘missing’ or ‘other’ (e.g., gender: non-binary/non-conforming) or merged with another relevant group (e.g., single/divorced/separated/widowed were merged to other). ‘Discrimination’ was ascertained from the binary question: “In the last 5 years, have you ever felt discriminated working as an IMG in Australia?”

Items within the mental health instruments were recoded for analysis. Firstly, items within the Wellbeing and K6 instruments were recoded to provide individual sum totals. Next, the K6 items were recoded to provide individual values which were dichotomized to high risk of having serious psychological distress (if total K6 score = 19–30 inclusive) or low risk of having serious psychological distress (if total K6 score ≤ 18). McDonald’s Omega coefficients were calculated to measure internal consistency of these instruments. The single burnout question was dichotomized to report any level of burnout (options [3] *“Sometimes I am stressed and consider myself to be burned out*,” [4] *“I am definitely burning out and have one or more symptoms of burnout, such as physical and emotional exhaustion,”* [5] *“The symptoms of burnout that I’m experiencing won’t go away- I think about frustration at work a lot,”* [6] *“I feel completely burned out and often wonder if I can go on- I am at the point where I may need some changes or may need to seek some sort of help”*) and report of no burnout (options [1] *“I enjoy my work and have no symptoms of burnout” and* [2] *“Occasionally I am under stress and I don’t always have as much energy as I once did, but I don’t feel burned out”*).

The proportion of doctors self-reporting risk of psychological distress and burnout were described using *N* (%), and for the Wellbeing score and total K6, means (SD) and ranges are reported. Crude associations between mental health variables and factors of interest were explored via *T*-test (Wellbeing) and Chi-squared test (K6 and Burnout). ANOVA (for Wellbeing) or Chi-squared test (for K6 and Burnout) were used when comparing 2 + groups of variables (e.g., Registration status’). Spearman’s correlation tested the Wellbeing variable against Age as a continuous variable. Analysis was conducted on complete cases, assuming missing data was MCAR (Missing Completely at Random). Missing data was excluded in testing associations.

## Results

Sample demographics: Of the 286 participants who started the survey, 199 completed the Wellbeing instrument, 191 completed the K6 instrument, and 181 completed the Burnout instrument, giving a completion rate of 63.29%–69.58%. Socio-demographic data for the sample are presented in Table 1. Participants came to Australia with primary medical qualifications from a total of 46 countries. 44 native languages were reported by the sample participants. The top 3 native languages reported by participants were English (61/241, 25.3%), Hindi (19/241, 7.9%), Arabic (16/241, 6.6%). The top 3 PMQ countries of participants were India, accounting for19.6% of participants (45/230), followed by the UK (33/230, 14.4%) and China (15/230, 6.5%). Participants from each state/territory participated in the survey. The majority of participants reported being located in New South Wales (116/223; 52%), Victoria (42/223; 18.8%) and Queensland 28/223 (12.6%). Over 70% of participants reported being located in metropolitan areas and about a third worked in rural and remote regions.

**TABLE 1:** Socio-demographic data of participants (*N* = 286).

Variable	Category	*N* (%)
** Age**	Years	40.4 (8.9)*
	*Missing*	*51*
** Gender**	Male	71 (28.3%)
	Female	179 (71.3%)
	Non-binary/non-conforming	1 (0.4%)
	*Missing*	*35*
** Marital status**	Married/*de facto*	197 (81.4%)
	Never married	33 (13.6%)
	Divorced/separated/widowed	12 (5.0%)
	*Missing*	*44*
** Native language**	English	61 (25.6%)
	Other	177 (74.4%)
	*Missing*	*48*
** Religion**	No religion (agnostic/atheist/non- religious/secular)	52 (21.8%)
	Religion	187 (78.2%)
	*Missing*	*47*
** PMQ country**	Competent authority pathway (UK, Ireland, USA, Canada, NZ)	54 (23.5%)
	Other	176 (76.5%)
	*Missing*	*56*
** Employment status**	Full-time	151 (65.9%)
	Part-time	56 (24.5%)
	Not working (e.g., unemployed, voluntary work, on extended leave, retired)	22 (9.6%)
	*Missing*	*57*
** Currently undertaking training**	Yes	49 (21.3%)
	No	181 (78.7%)
	*Missing*	*56*
Current region of work*	Metropolitan	152 (71.4%)
	Rural	65 (30.5%)
	Remote	7 (3.3%)
	Mixed- e.g., metropolitan and rural; or regional and remote	*11 participants reported working across >1 region
	*Missing*	*73*
** Registration status**	Full	125 (57.6%)
	Provisional/limited/non-practicing	65 (30.0%)
	Not registered	27 (12.4%)
	*Missing*	*65*
** Discrimination in the last 5 years**	Yes	121 (59.3%)
	No	83 (40.7%)
	*Missing*	*82*

*Mean (SD).

Mental health findings: Three instruments were used to explore three facets of mental health: wellbeing, likelihood of serious psychological distress and burnout. The distribution of cases across factors of interest are shown in Tables 2–4; results with a significant *p*-value area indicated with an Asterix. McDonald’s Omega coefficients were used to assess the internal validity of the instruments. The values were > 0.9 for the individual Wellbeing (0.94) and K6 (0.90) instruments, indicating excellent internal validity. The McDonald’s Omega coefficient for the three outcome instruments combined was also high (0.93), indicating that these instruments measured a similar underlying construct when combined.

**TABLE 2:** Association between Wellbeing and independent variables.

Wellbeing item; *N* = 199/286					
		*n*	Mean (/100) (95% CI)	*P*-value	Mean difference (95% CI)
**Gender**	Female	145	52.83 (49.07 – 56.59)	0.11^a^	−5.89 (−13.18 to −1.40)
	Male	53	58.72 (52.26 – 65.17)		
*Missing*	1				
**Married**	Married or *de facto*	155	55.69 (52.19 – 59.19)	0.07^a^	−7.91 (−16.34 to −0.51)
	Other	36	47.78 (38.60 – 56.95)		
*Missing*	8				
**Native language**	English	56	56.93 (50.61 – 63.25)	0.38^a^	−3.23 (−10.42 to −3.95)
	Other	143	53.71 (49.75 – 57.56)		
*Missing*	0				
**Religion**	No religion	46	58.70 (52.16 – 65.24)	0.20^a^	−5.04 (−12.76 to −2.69)
	Religion	140	53.66 (49.75 – 57.56)		
*Missing*	13				
**PMQ country**	Competent authority pathway	51	58.51 (51.84- 65.18)	0.11^a^	−6.03 (−13.36 to −1.29)
	Other	143	52.48 (48.77 – 56.18)		
*Missing*	5				
**Employed**	Working part or full-time	180	54.87 (51.50 – 58.24)	0.35^a^	−5.18 (−16.10 to −5.74)
	Non-working	19	49.68 (38.36 – 61.00)		
*Missing*	0				
**Training**	Yes	41	52.29 (45.35 – 59.23)	0.52^a^	2.65 (−5.41 to −10.70)
	No	155	54.94 (51.20 – 58.68)		
*Missing*	3				
**Work region**	Rural/remote/mixed	60	52.47 (47.30 – 57.63)	0.36^a^	3.24 (−3.76 to −10.24)
	Metropolitan only	123	55.71 (51.48 – 59.93)		
*Missing*	16				
**Registration**	Full	115	59.93	0.83^b^	
	Provisional/limited/non-practicing	56	46.21		
	None	23	48.37		
*Missing*	5				
**Discrimination in last 5 years**	Yes	113	46.62 (42.55 – 50.68)	<0.001^a***^	19.28 (13.26–25.30)
	No	82	65.90 (61.52–70.28)		
*Missing*	4				

a) P-value from t-test of total Wellbeing across categories of the independent variables;

b) P-value from ANOVA test of independent variable with total Wellbeing.

*** indicates *p* < 0.001.

**TABLE 3:** Association between K6 and independent variables.

K6 (Psychological distress) item; *N* = 191/286					
		n	Low risk score: i.e., K6: 0–18 *n* (% total)	High risk score: i.e., K6: 19–30 *n* (% total)	*P*-value
**Gender**	Female	138	113 (81.88)	25 (18.12)	0.17
	Male	51	46 (90.2)	5 (9.8)	
*Missing*	2				
**Marital status**	Married or *de facto*	148	126 (85.14)	22 (14.86)	0.22
	Other	34	26 (76.47)	8 (23.53)	
*Missing*	9				
**Native language**	English	53	44 (83.02)	9 (16.98)	0.76
	Other	138	117 (84.78)	21 (15.22)	
*Missing*	191				
**Religion**	No religion	44	38 (86.36)	6 (13.64)	0.82
	Religion	133	113 (84.96)	20 (15.04)	
*Missing*	14				
**PMQ country**	Competent authority pathway	48	41 (85.42)	7 (14.58)	0.81
	Other	137	115 (83.94)	22 (16.06)	
*Missing*	6				
**Employed**	Working part or full-time	174	148 (85.06)	26 (14.94)	0.29
	Non-working	16	12 (75.00)	4 (25.00)	
*Missing*	1				
**Training**	Yes	37	33 (89.19)	4 (10.81)	0.43
	No	150	126 (84.00)	24 (16.00)	
*Missing*	4				
**Work region**	Rural/remote/mixed	55	50 (90.91)	5 (9.09)	0.12
	Metropolitan only	120	98 (81.67)	22 (18.33)	
*Missing*	16				
**Registration**	Full	110	96 (87.27)	14 (12.73)	0.18
	Provisional/limited/non-practicing	55	47 (85.45)	8 (14.55)	
	None	21	15 (71.43)	6 (28.57)	
*Missing*	5				
**Discrimination in last 5 years**	Yes	108	87 (80.56)	21 (19.44)	0.09
	No	78	70 (89.74)	8 (10.26)	
*Missing*	5				

**TABLE 4:** Association between burnout and independent variables.

Burnout item; *N* = 181/286					
		*n*	Burnout (% total)	No burnout *n* (% total)	*P*-value
**Gender**	Female	129	64 (49.61)	65 (50.39)	0.16
	Male	50	19 (38)	31 (62)	
*Missing*	2				
**Marital status**	Married or *de facto*	140	62 (44.29)	78 (55.71)	0.55
	Other	34	17 (50.00)	17 (50.00)	
*Missing*	7				
**Native language**	English	52	25 (48.08)	27 (51.92)	0.78
	Other	129	59 (45.74)	70 (54.26)	
*Missing*	0				
**Religion**	No religion	41	19 (46.34)	22 (53.66)	0.86
	Religion	125	56 (44.80)	69 (55.20)	
*Missing*	15				
**PMQ country**	Competent authority pathway	47	22 (46.81)	25 (53.19)	0.85
	Other	128	62 (48.44)	66 (51.56)	
*Missing*	6				
**Employment status**	Working part or full-time	175	82 (46.86)	93 (53.14)	0.76
	Non-working	5	2 (40.00)	3 (60.00)	
*Missing*	1				
**Training**	Yes	40	22 (55.00)	18 (45.00)	0.21
	No	137	60 (43.80)	77 (56.20)	
*Missing*	4				
**Work region**	Rural/remote/mixed	55	27/55 (49.09)	28/55 (50.91)	0.41
	Metropolitan only	111	47/111 (42.34)	64/111 (57.66)	
*Missing*	15				
**Registration**	Full	110	52 (47.27)	58 (52.73)	0.89
	Provisional/limited/non-practicing	52	24 (46.15)	28 (53.85)	
	None	15	8 (53.33)	7 (46.67)	
*Missing*	4				
**Discrimination in last 5 years**	Yes	110	69 (62.73)	41 (37.27)	<0.001***
	No	67	14 (20.9)	53 (79.10)	
*Missing*	4				

***Indicates *p* < 0.001.

Wellbeing instrument: 199 participants completed the WHO 5-item Wellbeing instrument. The calculated mean score for participants was 54.6/100 [SD 23.18; median score of 80/100 recorded by 27/199 participants; range: 0–100].

### Associations with wellbeing instrument

The Spearman’s Rho test (*n* = 194) showed a weak correlation (0.07) between age and Wellbeing scores which was not statistically significant (*p* = 0.30). There were no statistically significant results when assessing association between the Wellbeing variable and gender, marital status, English as a native language, religion, country of PMQ, employment status, training status, registration status or work region variables (see Table 2 below).

Notably, those who had reported experiences of discrimination in the last 5 years had an average Wellbeing score 19.28 points below those who had not reported discrimination (46.62 vs. 65.90); a significant result: *p* < 0.001.

Kessler 6 (K6) instrument: 191 participants completed the K6 instrument. The mean K6 score was 12.8 [SD 5.47; range: 6–30]. 161/191 (84.3%) recorded a score between 0 and 18, indicating a low likelihood of serious psychological distress. 30/191 (15.7%) recorded a score between 19 and 30, indicating a high likelihood of serious psychological distress.

### Associations with K6 instrument

There was no significant difference between K6 low and high-risk results and Age (*n* = 186) [Low risk K6 score vs. High risk K6 score respectively; *n* = 157 vs. 29; mean age years = 40.87 (39.48–42.26) vs. 40.62 (36.64–44.60); mean difference = 0.25 (−3.37 to 3.87)]; *p*-value 0.89. There was also no significant difference seen between K6 results and gender, marital status, English as a native language, religion, country of PMQ, employment, training, registration status nor work region (see Table 3 below).

There was some evidence of a direction of effect when tabulating the discrimination variable against the K6 instrument, although the *p*-value did not reach statistical significance. Higher K6 scores were seen with a higher percentage of participants reporting discrimination over the past 5 years (21/108; 19.44%), compared to those who did not report discrimination over the past 5 years (8/78; 10.26%; *p* = 0.09).

Burnout instrument: 181 participants completed the single-item instrument for physician burnout. The mean Burnout score was 2.69, SD 1.75; median score 2: *“Occasionally I am under stress, and I don’t always have as much energy as I once did, but I don’t feel burned out”* (69/181, 38.12% participants). 97/181 (53.6%) participants recorded a score indicating no burnout and 84/181 (46.4%) participants recorded a score indicating some level of burnout.

### Associations with burnout instrument

There was no significant difference between Burnout and Age (*n* = 176) [Burnout vs. No Burnout respectively: *n* = 78 vs. 96; mean age years = 41.37 (39.60 – 43.14) vs. 40.92 (38.95–42.88); mean difference = −0.46 (−3.14 to 2.23)]; *p*-value 0.74. There was also no statistical difference seen between the Burnout results and gender, marital status, English as a native language, religion, country of PMQ, employment, training, registration status nor work region (see Table 4 below).

A very statistically significant result was seen when the main discrimination variable was tabulated against the burnout variable. IMGs reporting discrimination in the last 5 years were more likely to report burnout (69/110; 62.73%) than those who had not reported discrimination in the past 5 years (14/67; 20.9%); *p* < 0.001.

## Discussion

Our study provides recent data exploring the prevalence of mental health outcomes among a sample of IMGs in Australia. We found a broad range of wellbeing scores, with a reassuring mean and median sitting in the mid to high range. Similarly, we found only a minority of IMGs recorded high K6 scores indicating that the likelihood of serious psychological distress was low in the majority of participants. Near equal proportions of participants reported burnout and no burnout. However, as a group, those who reported poorer mental health outcomes in Wellbeing and Burnout were also more likely to have reported experiencing discrimination in the last 5 years; a significant finding (*p* < 0.001). The failure of the *p*-value to reach statistical significance in the instance of the binary discrimination variable vs. K6 outcome (*p* = 0.09) may be a consequence of our small sample size not achieving enough power to demonstrate a difference.

The stressors of relocation, acculturation, language, career and other psychosocial issues affecting IMGs have been broadly described in qualitative studies (16, 17, 29). Psychological stressors such as perceptions around finances, lack of family support, cultural isolation, autonomy and mental workload have been implicated as contributors to impaired IMG wellbeing (30, 31). A recent large longitudinal Australian study into wellbeing of medical graduates indicated generally high levels of wellbeing among all doctors in the study (including IMGs) although IMGs demonstrated lower scores when compared to domestic graduates (30). On the other hand, a 2013 national Australian mental health survey found that when compared to domestic graduates, IMGs reported being significantly less stressed by work stressors 20.2% cv 26.7% (e.g., workload, making correct decisions and responsibility), but more highly stressed by racism (4.3% cv 0.74%) (4). A recently published mixed methods study conducted by the authors found that IMGs reported a range of physical and mental health sequelae resulting from experiences of inequitable treatment in the workplace (15). Participants reported symptoms of depression and anxiety- low mood, lack of self-worth, low confidence, stress and fear in response to a range of inequitable experiences, such as bullying and institutional discrimination (15).

We found no studies directly investigating depression, anxiety or suicide. Atri’s 2009 USA study (32) of 108 IMGs (who were non-English native speakers) psychiatry residents found a sizeable proportion (> 92%) of participants recording high K6 scores indicating high likelihood of serious psychological distress; a figure which differs significantly from our findings. We found no association between work region and mental health outcomes in our study, but there is evidence elsewhere that health professionals working in rural or remote areas of Australia are vulnerable to poorer mental health due to higher expectations of independence and lower access to support networks (4).

Of the few studies we found exploring burnout in IMGs, we found similar rates (∼30%–50%) when compared to our study (33, 34). Numerous studies have consistently reported lower levels of burnout in IMGs when compared to domestic graduates, by up to 50% (33–35). Shakir also found higher levels of resilience among IMGs when compared to their domestic counterparts in USA, suggesting that resilience may protect against burnout (34).

Various levels of support have been recommended to protect IMG health including comprehensive induction, onboarding and mentoring (18, 36). Mentoring, or buddying programs, along with acculturation interventions such as language courses and education into the host country’s culture and history have also been suggested (18, 32). Future studies are necessary to better understand how institutions and policy makers can holistically support IMGs, by directly engaging IMGs in research. By asking about their lived experiences, perceptions and opinions about actionable solutions, future researchers have the opportunity to gather and present powerful consumer-led ideas for change.

### Strengths and limitations

Other than our previous studies (14, 15), we have not found any other research directly attempting to investigate a link between discrimination and mental health outcomes for IMGs. This cross-sectional study appears to be the first of its kind to identify that discrimination may be implicated as a factor associated with impaired wellbeing and higher burnout rates in IMGs. This supports the growing evidence indicating racial discrimination as a determinant of health, impacting both mental and physical health outcomes in various ethnic minority groups (3, 37).

It is challenging to determine if our sample group’s demographics accurately reflect the broader population of IMGs in Australia, as there is no publicly available repository providing this information, either for those working clinically or for those in non-clinical roles or who have left the medical field.

Our study is limited by the small study size and study design, including self-selection bias. As this study was purposefully designed as an exploratory investigation, a convenience sampling approach was used to facilitate recruitment. While this limits generalizability, it allowed us to gather preliminary insights into an under-researched population. The study findings are helpful in triggering and informing future studies with larger sample sizes. Furthermore, to our knowledge the single-item burnout instrument, although validated against physicians in international studies, it has not been validated against IMGs *per se*. Although the brevity of the tool is beneficial for user-friendliness, it limits understanding of the complexities of Burnout, and therefore the authors would recommend a more comprehensive Burnout tool to be used in future studies.

The lengthiness of the survey may have contributed to uptake. However, our participant number (286) is remarkable given the absence of incentives provided to participants, indicating a genuine willingness for participants to freely participate and share their experiences. Our data precision was slightly lower than expected due to the loss of some participant numbers as the survey progressed. There is potential for bias as missing data was excluded in analysis.

Given the inability to identify the true number of IMGs in Australia, a power calculation was not possible. Although our choice for short instruments was beneficial to survey user-friendliness, it may have resulted in underreporting, particularly in the case of burnout. Regardless, our study provides rich descriptive data not evidenced elsewhere. The temporal relationship between the main discrimination variable and mental health outcomes may be reconsidered in future studies. The scarcity of existing research in this field, also makes thorough interpretation of our work difficult. Despite these limitations, our study provides insight into a variety of outcomes experienced by IMGs. Further evaluation in larger, robust studies would be valuable to better map demographics and assess more complex relationships such as significance and causality by undertaking more advanced statistical modeling techniques.

## Conclusion

We explored self-reports of wellbeing, rates of psychological distress and burnout (emotional exhaustion component), amongst IMGs in Australia. Participants completing the Wellbeing instrument reported a broad range of scores, with reassuring mid to high range scores for the mean and median respectively. The majority of participants completing the K6 instrument indicated a low likelihood of serious psychological distress. Almost half of the participants completing the single-item burnout instrument indicated some level of burnout.

We identified statistically significant associations between reported adverse mental health outcomes in the Wellbeing and Burnout instruments and reports of discrimination in the last 5 years. Exploring the prevalence of mental health outcomes among this IMG sample has been valuable in better understanding the burden of stress among this largely overlooked community. However, further study in larger and more robust studies is necessary to confirm and explore these preliminary findings. Further exploration of mental health outcomes and challenges unique to IMGs is warranted, in order to support and sustain this global workforce who are vital for health care systems and society.

## Data Availability

All study data is available within the text and tables of this manuscript. Ethical restrictions imposed by the University of Newcastle Human Research Ethics Committee prevents public sharing of raw datasets, to protect the privacy and confidentiality of the small sample population.
